# Sodium, potassium intake, and all-cause mortality: confusion and new findings

**DOI:** 10.1186/s12889-023-17582-8

**Published:** 2024-01-15

**Authors:** Donghao Liu, Yuqing Tian, Rui Wang, Tianyue Zhang, Shuhui Shen, Ping Zeng, Tong Zou

**Affiliations:** 1 Department of Cardiology, Beijing Hospital, National Center of Gerontology: Institute of Geriatric Medicine, Chinese Academy of Medical Sciences, Beijing, 100730 People’s Republic of China; 2 Beijing Hospital, National Center of Gerontology, Institute of Geriatric Medicine, Chinese Academy of Medical Sciences and Peking Union Medical College, Beijing, 100730 People’s Republic of China; 3 Beijing Hospital, Institute of Geriatric Medicine, Peking University Fifth School of Clinical Medicine, Beijing, China

**Keywords:** Sodium intake, Potassium intake, Sodium–potassium ratio, All-cause mortality

## Abstract

**Background:**

The World Health Organization (WHO) has established recommended daily intakes for sodium and potassium. However, there is currently some controversy regarding the association between sodium intake, potassium intake, the sodium-to-potassium ratio, and overall mortality. To assess the correlations between sodium intake, potassium intake, the sodium-to-potassium ratio, and overall mortality, as well as the potential differences in sodium and potassium intake thresholds among different population groups, we analyzed data from NHANES 2003–2018.

**Methods:**

NHANES is an observational cohort study that estimates sodium and potassium intake through one or two 24-h dietary recalls. Hazard ratios (HR) for overall mortality were calculated using multivariable adjusted Cox models accounting for sampling design. A total of 13855 out of 26288 participants were included in the final analysis. Restricted cubic spline analyses were used to examine the relationship between sodium intake, potassium intake, and overall mortality. If non-linearity was detected, we employed a recursive algorithm to calculate inflection points.

**Results:**

Based on one or two 24-h dietary recalls, the sample consisted of 13,855 participants, representing a non-institutionalized population aged 40–80 years, totaling 11,348,771 person-months of mean follow-up 99.395 months. Daily sodium intake and daily potassium intake were inversely associated with all-cause mortality. Restrictive cubic spline analysis showed non-linear relationships between daily sodium intake, potassium intake, sodium–potassium ratio, and total mortality. The inflection point for daily sodium intake was 3133 mg/d, and the inflection point for daily potassium intake was 3501 mg/d, and the inflection point for daily sodium–potassium ratio intake was 1.203 mg/mg/d. In subgroup analyses, a significant interaction was found between age and high sodium intake, which was further confirmed by the smooth curves that showed a U-shaped relationship between sodium intake and all-cause mortality in the elderly population, with a inflection point of 3634 mg/d.

**Conclusion:**

Nonlinear associations of daily sodium intake, daily potassium intake and daily sodium–potassium ratio intake with all-cause mortality were observed in American individuals. The inflection point for daily sodium intake was 3133 mg/d. And the inflection point for daily sodium intake was 3634 mg/d in elderly population. The inflection point for daily potassium intake was 3501 mg/d. The inflection point for daily sodium–potassium ratio intake was 1.203 mg/mg/d, respectively, A healthy diet should be based on reasonable sodium intake and include an appropriate sodium-to-potassium ratio.

**Supplementary Information:**

The online version contains supplementary material available at 10.1186/s12889-023-17582-8.

## Introduction

Sodium and potassium are two essential minerals that play a critical role in maintaining normal physiological functions and electrolyte balance. Sodium intake has a dual impact on mortality. On one hand, it is evidenced [1, 2] that restricting sodium intake can significantly lower blood pressure, particularly in individuals with hypertension. On other hand, increasing salt intake to stimulate appetite and improve nutritional status can reduce mortality in some special population [3]. A plenty of dietary guidelines advocate for a reduction in sodium intake to 2300 mg per day(mg/d), and the World Health Organization (WHO) even advises curtailing adult sodium intake to less than 2000 mg/d [4]. There is currently a debate [5–17] about whether adopting a low-sodium diet can reduce the risk of mortality in the entire population. The conclusion that a low-sodium diet leads to a decrease in mortality may only be applicable to specific populations [15, 16], and there may be differences in the sodium intake thresholds among different population groups. Concerning potassium consumption, current research finds a relationship between increased daily potassium intake and a decreased risk of cardiovascular mortality [18], which may also be interconnected with sodium intake. One study [19] discerned that the risk associated with high sodium intake changes with potassium intake. When potassium intake decreases, it leads to sodium retention and elevated blood pressure. The WHO proposes a daily potassium intake of at least 90 mmol/L for the general population [20]. However, a descriptive study anchored on NHANES 2003–2008 [21] revealed that fewer than 2% of adults meet the WHO-recommended potassium intake. Whether high potassium intake can reduce the risk of all-cause mortality and whether there are differences in various populations remain understudied. Given that sodium and potassium do not individually affect changes in mortality, the sodium–potassium ratio is increasingly being acknowledged as a potential dietary monitoring metric. There is evidence to suggest that the sodium–potassium ratio may exhibit a stronger association with blood pressure outcomes than using sodium or potassium alone [22].

Our study harnessed data from NHANES (2003–2018) to examine the association between daily sodium intake, daily potassium intake, and the sodium–potassium ratio with all-cause mortality. The adoption of NHANES 24-h dietary recalls for intake estimation is preferred for several reasons. Primarily, it is deemed accurate [23] and can be facilely applied on a substantial scale. Furthermore, employing urinary sodium and potassium as investigatory markers could potentially introduce statistical inaccuracies due to non-standardized sampling methodologies [6].

## Methods

### Exposure variables

The primary exposure variables include self-reported daily sodium and potassium intake. NHANES participants partook in in-person interviews utilizing the Automated Multiple Pass Method (AMPM) 24-h dietary recall at mobile examination centers. Measurement aids, including cups, utensils, and measuring cups, were furnished during these interviews to facilitate accurate reporting of food consumption quantities [24]. Dietary interviews conducted face-to-face furnished estimations of the types and quantities of food ingested by the participants in the 24 h preceding the interview. From these metrics, the intake of assorted electrolytes was indirectly gauged. In our research, responses were retained only for the 'usual' category when asked to compare the food consumed on the prior day to a typical day. Sodium intake was categorized as per common classification [4, 25] as 'low sodium intake' (< 2300 mg/day), 'normal intake' (≥ 2300 mg/day and < 4600 mg/day), and 'high sodium intake' (≥ 4600 mg/day). Potassium intake was analyzed using the population's tertiles, with T1 (< 2157 mg/day), T2 (≥ 2157 mg/day and < 3000 mg/day), and T3 (≥ 3000 mg/day). For variable transformation, the sodium–potassium ratio was construed as the proportion of sodium intake to potassium intake and analyzed using the tertiles of the study population: T1 (< 1.07 mg/mg/day), T2 (≥ 1.07 mg/mg /day and < 1.44 mg/mg/day), and T3 (≥ 1.44 mg/mg /day). These variables were treated as continuous for restricted cubic spline analysis.

### Study population

The analyzed data is derived from NHANES 2003–2016, which employs a sophisticated, stratified, multi-stage probability sampling technique to amass health and nutrition data from a representative, non-institutionalized U. S. population. The dataset amalgamates household interviews and physical examinations, inclusive of in-person 24-h dietary recall interviews conducted at Mobile Examination Centers (MECs). Proficient interviewers leverage Computer-Assisted Personal Interviewing (CAPI) systems to gather demographic data and characteristics during household and MEC visits. Information pertaining to age, sex, ethnicity, marital status, income, smoking status, alcohol consumption, potassium consumption, total calorie intake, and sodium intake is procured from the household interview data. Meanwhile, Body Mass Index (BMI) data is acquired from the examination data. A total of 26,288 participants were included in the final analysis, after excluding those with inadequate memory recall (*n* = 9043), missing education status (*n* = 14), missing PIR (*n* = 1451), missing smoking status (*n* = 65), missing drink levels (*n* = 923), missing medical conditions (*n* = 146), missing BMI (*n* = 142), missing laboratory examination (*n* = 601), missing marriage status (*n* = 6), missing death data (*n* = 14), missing physical activity (*n* = 28). A total of 13,855 individuals were ultimately included in the study. (Supplement Figure 1).

### Definition of results

The primary outcome is all-cause mortality, defined as the time from baseline examination to death from any cause, using data obtained from NCHS Surveys Linked to NDI Mortality Data.

### Covariates

The multivariate analysis adjusted for an array of covariates, including age, sex, race, presence of diabetes, hypertension, cardiovascular disease (CVD), physical activity, body mass index (BMI), estimated glomerular filtration rate (eGFR), smoking habits, alcohol consumption, income level, marital status, activity level, and caloric intake. Marital status was determined based on whether the respondent lived alone or cohabitated with a partner. The poverty income ratio (PIR), a measure of income level, was computed by dividing household income by the poverty guidelines specific to the survey year, which vary depending on family size and geographic location. In this study, PIR was used to delineate two income brackets: low (PIR < 1.3) and high (PIR ≥ 1.3), thereby serving as a proxy for socioeconomic status based on eligibility for the Supplemental Nutrition Assistance Program (SNAP) benefits [26]. Educational attainment was dichotomized into two groups: those holding a college or Associate's degree and those below this threshold. Smoking status was bifurcated into smoking and non-smoking. Alcohol consumption was classified according to the frequency of alcohol intake per month: never, 1–10 times/month, or over 10 times/month [27]. Given that more than a third of Americans are obese [28], BMI was segmented into three categories: normal (< 24.9 kg/m^2^), overweight (25–30 kg/m^2^), and obese (≥ 30 kg/m^2^). Participants were considered physically inactive if they responded "no" to all activity-related queries in the questionnaire, and active otherwise.

Participant medical histories were self-reported. In the context of CVD, a participant was considered a CVD patient if they responded affirmatively to the question, "Has a doctor or healthcare professional ever diagnosed you with coronary heart disease/angina, also referred to as chest pain/heart attack (also known as myocardial infarction)/stroke/congestive heart failure (CHF)?" The diagnostic criteria for diabetes included any of the following: a self-reported medical history of diabetes diagnosis, HbA1c (%) exceeding6.5, random blood glucose (mmol/L) of 11.1 or above, 2-h OGTT (Oral Glucose Tolerance Test) blood glucose (mmol/L) of 11.1 or above, or the use of diabetes medication or insulin. Hypertension diagnostic criteria were defined by a self-reported medical history of diagnosed hypertension, systolic blood pressure exceeding140 mmHg at the MEC, or the use of antihypertensive medication. The CKD-EPI equation [29] was utilized to calculate the creatinine clearance rate in this study. An eGFR below 60 ml/min denoted the chronic kidney disease (CKD) population.

### Statistical analysis

Our analysis incorporated the NHANES sample weights and accommodated the intricate multi-stage cluster survey design. We represented continuous variables using their mean and standard deviation (SD), while categorical variables were expressed in counts and percentages. and the chi-square test was used for categorical variables. To explore the relationship between variables and all-cause mortality, we conducted event-time analysis. Single-factor and multi-factor analyses were performed using the Cox proportional hazards regression model. We validated the proportional hazards assumption of the variables using Schoenfeld residuals. To address the issue of multiple testing, the Benjamini–Hochberg procedure was applied to control the false discovery rate (FDR). In trend tests, the median of each group was treated as a continuous variable in the model, and FDR correction was applied to adjust for multiple tests. Variance inflation factors (VIF) were calculated to assess multicollinearity among variables in the Cox model. VIF values below 10 indicated a low likelihood of multicollinearity affecting the estimates [30]. Subsequently, a series of sensitivity analyses were conducted to ensure the robustness of the data analysis, including likelihood ratio tests using models with and without interaction terms for interaction testing.

To investigate the relationships between daily sodium intake, potassium intake, and the sodium–potassium ratio with all-cause mortality, we constructed restricted cubic spline analyses for sodium intake, potassium intake, and the sodium–potassium ratio within a multivariable framework. This allowed us to examine the non-linear relationships between these factors and all-cause mortality. We further explored the changes in hazard ratio (HR) and 95% confidence intervals (95% CI) of sodium intake, potassium intake, and the sodium–potassium ratio relative to reference points. A population reference point of 2300 mg was used for sodium intake, with the first tertile serving as reference points for potassium intake and the sodium–potassium ratio. If non-linearity was detected, we employed a recursive algorithm to calculate inflection points.

All analyses were conducted according to CDC guidelines using R software (version4.2.3).

## Result

In our study, a total of 17,245 subjects were included, and after excluding missing values, we had 13,855 remaining participants, representing a weighted target population of 11,348,771 individuals. The mean follow-up duration was 99.395 (52.825) months, and there were 1906 deaths. The demographic characteristics, prevalence of diabetes, hypertension, and CVD of the participants after excluding missing values in the weighted population (see Supplement Table 1) were generally similar to the overall population. Only minor differences were observed in terms of sex, race, PIR, sodium intake, potassium intake, and calorie intake.

Supplementary Table 2 presents the demographic characteristics based on different daily sodium intake groups. This table reports various demographic and health-related factors, with significant differences observed between different daily sodium intake groups. Supplementary Table 3 displays the demographic characteristics based on different daily potassium intake groups. Individuals with higher daily sodium or potassium intake tended to be younger, male, from married households, and with higher income, among other factors.

### Sodium intake

In Table 1, we designed three Cox regression models to investigate the independent effect of daily sodium intake on all-cause mortality. After adjusting for multiple factors, including sex, age, education, race, poverty income ratio, smoking, drinking, hypertension, diabetes, cardiovascular disease, BMI, eGFR, physical activity, dietary calorie intake, and potassium intake (model 2), the multivariable-adjusted HR and 95% CIs for all-cause mortality risk, from the lowest to the highest sodium intake levels (< 2300 mg/d, 2300-4600 mg/d, > 4600 mg/d), were 1.00 (reference), 0.79 (0.66, 0.95), and 0.69 (0.50, 0.96), respectively (P for trend = 0.013). In subsequent subgroup analyses, we found that high sodium intake(> 4600 mg/d), compared to low sodium intake(< 2300 mg/d), was associated with lower all-cause mortality in the younger population (Age >  = 40 & Age < 60), with a statistically significant interaction (P for interaction = 0.02) (see Supplement Figure 3). Additionally, high sodium intake, compared to low sodium intake, was related to lower all-cause mortality in the high-income population, although the *p*-value for interaction did not reach statistical significance (see Supplement Figure 3).

**Table 1:** The multicollinearity test was conducted for all variables in the models

Models^a^	Sodium Intake(mg)			*p*-value	q-value^b^	p-trend^c^
	**Low Intake**	**Normal Intake**	**High Intake**			
**Crude**	*ref*	0.74(0.61,0.89)	0.54(0.42, 0.70)	0.0015*	0.0045*	< 0.0001*
**Model 1**^**c**^	*ref*	0.82(0.69, 0.96)	0.74(0.57, 0.98)	0.030*	0.032*	0.016*
**Model 2**^**d**^	*ref*	0.79(0.66, 0.95)	0.69(0.50, 0.96)	0.032*	0.032*	0.013*
	**Potassium Intake(mg)**					
	**T1**	**T2**	**T3**			
**Crude**	*ref*	0.81(0.70, 0.95)	0.65(0.54, 0.78)	< 0.0001*	< 0.0001*	< 0.0001*
**Model 1**^**c**^	*ref*	0.93(0.81, 1.06)	0.78(0.66, 0.92)	0.012*	0.018*	0.0028*
**Model 2**^**e**^	*ref*	0.91(0.78, 1.06)	0.74(0.59, 0.93)	0.038*	0.038*	0.0091*
	**Ratio of sodium to potassium**					
	**T1**	**T2**	**T3**			
**Crude**	*ref*	0.93(0.76, 1.13)	1.07(0.89, 1.29)	0.34	0.225	0.5
**Model 1**^**c**^	*ref*	0.98(0.80, 1.19)	1.15 (0.97, 1.37)	0.15	0.225	0.13
**Model 2**^**f**^	*ref*	0.98(0.81, 1.20)	1.16(0.97, 1.37)	0.14	0.34	0.11

*indicates statistical significance

^a^The VIFs for each variable were less than 10

^b^q-values, false discovery rate (FDR)-corrected *p*-values for trend test, p-trend for trend test were obtained from the cox regression models by using the median of each group as a continuous variable. FDR corrections were performed to adjust for multiple tests

^c^Adjust for sex, age, education, race, poverty income ration, smoking, drinking, hypertension, diabetes, CVD, BMI, eGFR, physical activity

^d^Add the dietary calories intake, potassium intake in model 1

^e^Add the dietary calories intake, sodium intake in model 1

^f^Add the dietary calories intake in model 1

**Fig. 1:**
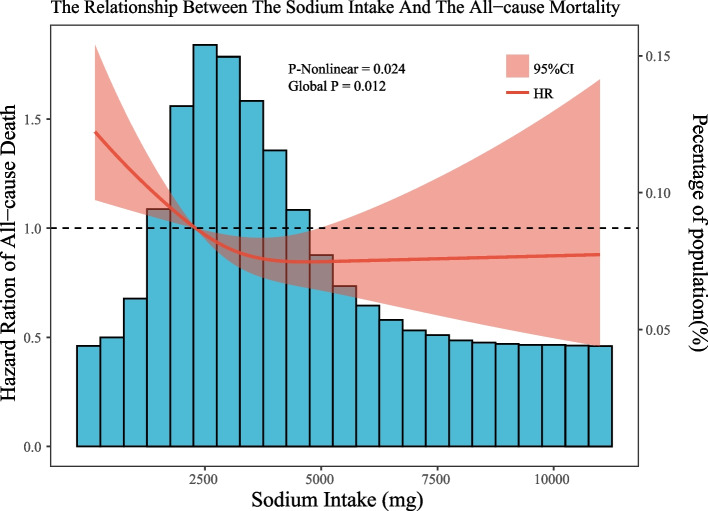
Dose–response relationships between sodium intake and all-cause mortality. Legend: The associations were adjusted for all covariates. The solid line and the ribbon represent the estimated HRs and its 95% CI

**Fig. 2:**
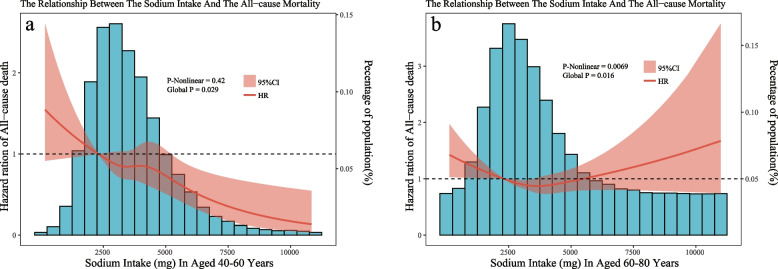
Dose–response relationships between sodium intake and all-cause mortality. Legend: The associations were adjusted for all covariates except age. The solid line and the ribbon represent the estimated HRs and its 95% CI. **a** represent the curve in age of 40–60, **b** represent the curve in age of 60–80

Using restricted cubic spline analyses, we discovered a non-linear association between daily sodium intake and all-cause mortality (Fig. 1). The inflection point for the recursive algorithm is calculated to be 3133 mg/d. When daily sodium intake was less than 3133 mg/d, there was a negative correlation with all-cause mortality. However, when it exceeded 3133 mg/d, sodium intake was not associated with all-cause mortality. Further exploration of the differences in sodium intake among different age groups was conducted by restricted cubic spline analyses for different age groups. The results revealed a negative linear correlation between daily sodium intake and all-cause mortality in the 40–60 age group, and a U-shaped correlation in the 60–80 age group, with the inflection point at 3634 mg/d (see Fig. 2).

### Potassium intake

In the univariate and multivariate Cox analyses conducted in Table 1, we obtained results similar to those of sodium intake. After adjusting for all covariates in model 3, the multivariable-adjusted hazard ratios (HR) and 95% confidence intervals (CIs) for all-cause mortality risk from the first tertile to the third tertile of intake levels (T1, T2, T3) were 1.00 (reference), 0.91 (0.78, 1.06), and 0.74 (0.59, 0.93), respectively (P for trend = 0.0091). Subgroup analysis results, as shown in Supplement Figure 4, Supplement Figure 5, revealed that even though the interaction did not reach a statistically significant level, in the subgroup with hypertension, high potassium intake (T3), compared to low potassium intake (T1), was associated with lower all-cause mortality among participants with hypertension, as well as among high PIR individuals.

**Fig. 3:**
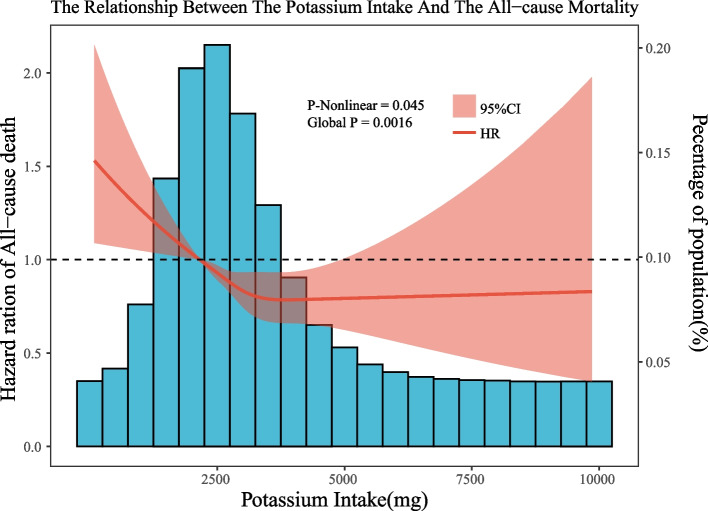
Dose–response relationships between potassium intake and all-cause mortality. Legend: The associations were adjusted for all covariates. The solid line and the ribbon represent the estimated HRs and its 95% CI. CI, confidence intervals; HR, Hazard ratio

Through restricted cubic spline, we identified a non-linear relationship between daily potassium intake and all-cause mortality (Fig. 3). The inflection point for the recursive algorithm is calculated to be 3501 mg/d. When daily potassium intake was less than 3501 mg/d, there was a negative correlation with all-cause mortality. However, when it exceeded 3501 mg/d, potassium intake was not associated with all-cause mortality.

### sodium–potassium ratio

**Fig. 4:**
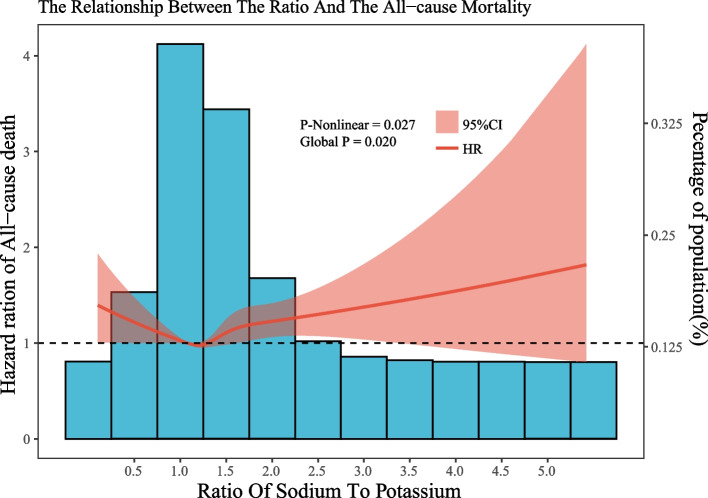
Dose–response relationships between Na:K and all-cause mortality. Legend: The associations were adjusted for all covariates. The solid line and the ribbon represent the estimated HRs and its 95% CI

In the univariate and multivariate Cox analyses conducted in Table 1, the sodium–potassium ratio was not associated with all-cause mortality. Similar results were obtained in subgroup analyses(see Supplement Figure 6, Supplement Figure 7). However, in the restricted cubic spline, we discovered a "U"-shaped correlation between the sodium–potassium ratio and all-cause mortality. The inflection point appeared at 1.203 mg/mg/d (Fig. 4).

## Discussion

The 24-h recall used in NHANES is less prone to bias compared to other dietary assessment methods, such as the food frequency questionnaire [31]. This notion is further reinforced by earlier studies including NHANES I [12], NHANES II [11], and NHANES III [32], which identified a comparable relationship between low sodium intake and a rise in all-cause mortality using the Food Frequency Questionnaires. These findings lend weight to our conclusions.

The analysis of the NHANES data in the present study demonstrated the associate between low sodium intake, low potassium intake, and a high sodium–potassium ratio with high risk in mortality in the univariate model among individuals aged 40 years and older. Intriguingly, this correlation remained significant even after accounting for other covariates. Nevertheless, a more detailed subgroup analysis uncovered further noteworthy findings.

### Sodium intake

The NHANES data provide us with a wide range of age-related information, allowing us to analyze the impact of age on the sodium-mortality risk relationship. This study found that for people aged 40 and above, daily sodium intake is negatively correlated with all-cause mortality. Our study uncovers a negative association between daily sodium intake and all-cause mortality for individuals over 40, corroborating findings from previous research conducted by Messerli FH et al. [5] and O'Donnell M et al. [6]. While a reduced sodium intake may diminish peak blood pressure and lower the likelihood of hypertension-related cardiovascular events, an exceedingly low intake can potentially stimulate the renin–angiotensin–aldosterone system (RAAS) [33, 34], impacting catecholamine and lipid metabolism and consequently raising the mortality risk. On conducting subgroup analyses, we observed a significant interaction between age and daily sodium intake. Consequently, we segmented the population based on age, which revealed a distinct negative relationship between daily sodium consumption and all-cause mortality among those aged 40–60 years. However, this correlation was not significant in the 60–80 age bracket, a finding that aligns with Kalogeropoulos AP et al.'s [15] conclusion from their ten-year longitudinal study of 2,642 individuals aged 71–80. Notably, post-adjustment for covariates, the dose–response curve in the 60–80 age segment suggested a U-shaped relationship between the HR and daily sodium intake. This implies that both excessively high or low sodium intake could escalate the risk of all-cause mortality within this demographic. In addition, our study indicates that, compared to a low-sodium diet, men derive greater benefits from normal or high sodium intake. Currently, there is no literature reporting the reasons for this; however, we hypothesize that this may be attributed to men having higher energy requirements than women. A correlation between sodium and energy intake is evident, and low sodium intake may suggest a lower nutritional status.

Our study also reveals a correlation between higher sodium intake and a younger age demographic. However, this differential did not correspond to an anticipated increase in all-cause mortality risk. Given the dynamics of sodium metabolism, this result may align with reality. Typically, younger individuals exhibit eGFR and robust renal regulation of sodium metabolism [35]. Thus, it is plausible to hypothesize significant variations in sodium metabolism inflection points across distinct age groups, partially accounting for the observed statistical differences in sodium intake among various age subgroups. For the elderly, the fragility of renal sodium metabolism regulation potentially escalates mortality risk associated with excessively high or low sodium intake. This necessitates further research to establish tailored daily sodium intake recommendations for older individuals. Additionally, we discerned a similar correlation curve between sodium intake and all-cause mortality in CKD patients, lending further credibility to our hypothesis (see Supplement Figure 8).

### Potassium intake

Our study identified a significant increase in all-cause mortality when daily potassium intake falls below 3500 mg, a result aligning with O'Donnell M et al.'s findings [6]. Prior studies [36, 37] highlight the integral role of potassium in blood pressure regulation, which our subgroup analysis, focused on hypertensive individuals, corroborates. Notably, we discovered that elevating daily potassium intake considerably mitigates all-cause mortality among hypertensive individuals (Supplement Figure 5). The antihypertensive effect of potassium, attributed to a variety of mechanisms, is well-recognized. These mechanisms encompass stimulation of natriuresis, enhancement of endothelial function, release of nitric oxide (NO), increased Na–K pump function, amplified membrane potassium channel activity, resulting in vasodilation and subdued sympathetic nervous system activity, thereby inducing arterial muscle relaxation [36].

### Sodium–potassium ratio

The sodium-to-potassium ratio is a significant factor in managing blood pressure and overall health. It is more strongly associated with blood pressure outcomes than either sodium or potassium alone, particularly in hypertensive adult populations [22]. What’s more? A study of healthy Greek adults found that food sodium intake was positively correlated with energy intake and food potassium intake [38]. The correlations observed between sodium or potassium intake and all-cause mortality may potentially reflect influences of nutritional status or the intensity of physical activity. The residual effects of these factors, which may not be entirely mitigated by statistical adjustments. Given the strong correlation between sodium and potassium intake and energy intake, employing the sodium–potassium ratio as a monitoring measure might be fitting, Current researches indicate that the sodium-to-potassium ratio, as an indicator of sodium and potassium intake, has been shown to be unrelated to total energy intake [39, 40]. From a statistical perspective, it is more effective in preventing biases introduced by reverse causation. Yet, there exist no formal recommendations within the United States advocating for such a use. Various studies indicate that cardiovascular event or composite risk heat maps demonstrate the lowest risk in the moderate sodium intake range of 3–5 g/d [41], accompanied by a higher potassium intake. The WHO stipulates the optimal sodium–potassium ratio as less than1 mmol/mmol (below 0.6 mg/mg) [42], but relevant research [43] validating the association between this ratio and all-cause mortality remains scarce. Current studies [43–45] concerning the sodium–potassium ratio primarily revolve around its connection with hypertension, with limited literature on its relation to all-cause mortality. Some research findings [46, 47] point towards a correlation between a high sodium–potassium ratio and the onset of cardiovascular diseases. In our study, in our study's restricted cubic spline model, we observed that as the sodium-to-potassium ratio exceeded 1.203 mg/mg/day, the overall mortality rate showed an increasing trend (see Fig. 4). Therefore, integrating our results with previous studies, we posit that maintaining a balanced sodium and potassium intake ratio within an appropriate range is vital for controlling all-cause mortality in the population. However, verifying the reliability of this conclusion necessitates further clinical research.

The findings of our study suggest that the WHO current recommendations on daily sodium and potassium intake for the general population might be oversimplified. The advisable amounts of daily sodium and potassium intake should ideally differ among various age groups and individuals with distinct underlying medical conditions [48]. Hence, the necessity for age-specific and individualized assessment techniques for evaluating sodium and potassium intake is evident. Nevertheless, focusing exclusively on a single nutrient indicator might yield inaccuracies, given that sodium and potassium intake can act as proxies for consumption patterns. Moreover, an excessively high or low sodium–potassium ratio might suggest poor dietary structure, resulting in an escalation in all-cause mortality. Recent reports [49, 50] discovered that, during 2015–2016, primary contributors to sodium intake were processed foods or store-bought foods with added sodium, such as deli sandwiches, poultry, or sodium-enhanced vegetables. In contrast, potassium intake predominantly originated from naturally low-sodium foods, such as plain milk, fruits, and vegetables, along with processed foods. Past researches [49, 51] indicate that individuals with higher income might maintain a more balanced daily dietary nutrient structure, thus achieving a sodium–potassium ratio closer to the optimum range. This could be one factor contributing to the disparity in all-cause mortality between high-income and low-income populations. However, even with similar daily dietary nutrient structures (similar sodium–potassium ratios), our study still identified substantial differences in all-cause mortality between high-income and low-income populations, as depicted in Supplement Figure 3, Supplement Figure 5, Supplement Figure 7, with the most conspicuous disparities occurring in the context of high sodium and potassium intake, This phenomenon could be attributed to disparities in healthcare access and conditions available to the two demographic groups in their daily lives.

Presently, the existing evidence surrounding dietary interventions is scant, with most conclusions extrapolated from the DASH study. Adjustments to other dietary structures, along with their beneficial or detrimental effects, cannot be exclusively ascribed to a singular electrolyte. Hence, additional controlled experiments are imperative to substantiate these conclusions [52].

There exist some limitations to the findings of this study. Firstly, the dietary data and medical conditions were self-reported, rendering them susceptible to recall and social desirability biases. Furthermore, our estimations do not account for the sodium present in salt added at the table, which represent 5–6% [53] of total intake, which may poorly reflect the variability in seasoning use. Secondly, the possibility of reverse causality could introduce a bias in the estimation of effects. Certain studies include populations with cardiovascular or other chronic diseases. Individuals from these groups might have been recommended to limit their sodium intake due to their potential diseases, thereby inadvertently associating reduced sodium intake with a marked rise in the risk of all-cause mortality among the low-level population [16].

Our findings highlighted that low sodium intake is associated with an increased overall mortality rate. Additional evidence is required to determine the potential benefits of restricted sodium intake for young individuals or those with normal kidney function. Secondly, the benefits of sodium and potassium intake vary among different populations, necessitating personalized recommendations for each group. Lastly, focusing solely on sodium and potassium intake may be one-sided. A healthy diet should be based on reasonable sodium intake and include an appropriate sodium-to-potassium ratio.

### Supplementary Information


**Additional file 1.**

**Additional file 2.**

**Additional file 3.**

**Additional file 4.**

**Additional file 5.**

**Additional file 6.**

**Additional file 7.**

**Additional file 8.**

**Additional file 9.**

**Additional file 10.**

**Additional file 11.**

## Data Availability

The datasets generated and analyzed in the current study are all available at NHANES website: https://www.cdc.gov/nchs/nhanes/index.htm.

## Abbreviations

BMI: Body Mass Index
CDC: Centers for Disease Control and Prevention
CKD: Chronic kidney disease
CVD: Cardiovascular disease
eGFR: Estimated glomerular filtration rate
HR: Hazard ratio
NHANES: National Health and Nutrition Examination Survey
PIR: Family poverty income ratio
VIF: Variance inflation factor
WHO: The World Health Organization
95% CI: 95% Confidence interval
