# Correction: Sodium, potassium intake, and all-cause mortality: confusion and new findings

**DOI:** 10.1186/s12889-024-18504-y

**Published:** 2024-04-18

**Authors:** Donghao Liu, Yuqing Tian, Rui Wang, Tianyue Zhang, Shuhui Shen, Ping Zeng, Tong Zou

**Affiliations:** 1 Department of Cardiology, Beijing Hospital, National Center of Gerontology: Institute of Geriatric Medicine, Chinese Academy of Medical Sciences, 100730 Beijing, People’s Republic of China; 2 Beijing Hospital, National Center of Gerontology, Institute of Geriatric Medicine, Chinese Academy of Medical Sciences and Peking Union Medical College, 100730 Beijing, People’s Republic of China; 3 Beijing Hospital, Institute of Geriatric Medicine, Peking University Fifth School of Clinical Medicine, Beijing, China

BMC Public Health (2024) 24:180

10.1186/s12889-023-17582-8

The original publication of this article contained an incorrect author name. The incorrect and correct information is listed in this correction article, the original article has been updated.

Incorrect

Ping Zen

Correct

Ping Zeng

