# Supplementary figures and images for: Sodium, potassium intake, and all-cause mortality: confusion and new findings
Source: BMC Public Health. 2024 Jan 15;24:180. doi: 10.1186/s12889-023-17582-8 (PMC10789005; doi:10.1186/s12889-023-17582-8)

Supplementary Figure 1. Flow chart of study participants

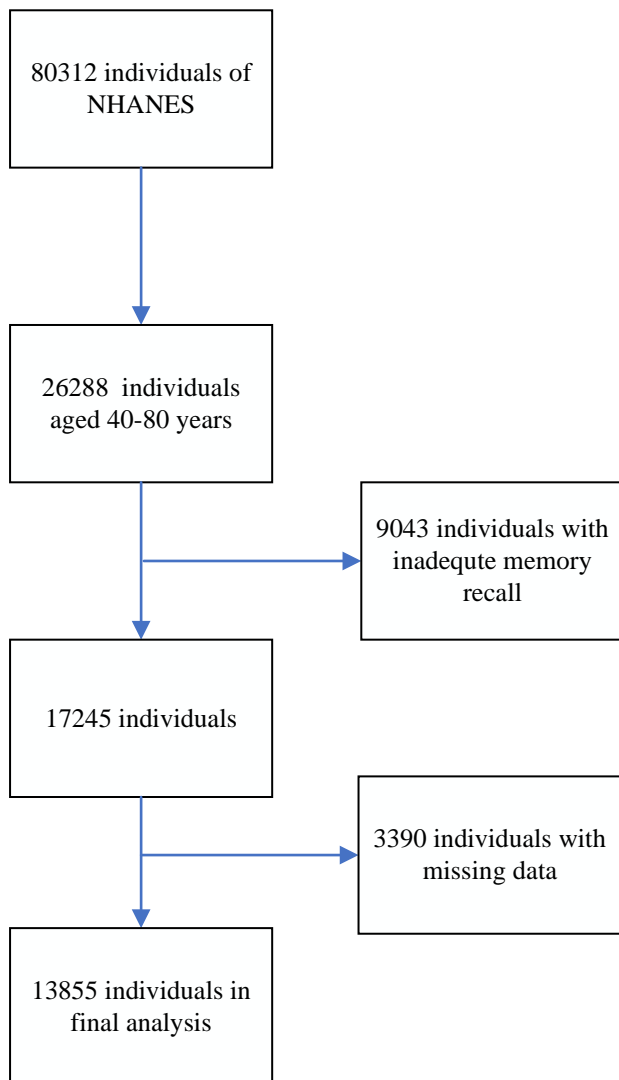

Supplement: Supplementary file 1 — Additional file 1. [file 12889_2023_17582_MOESM1_ESM.pdf]
