# Supplementary material for: Sodium, potassium intake, and all-cause mortality: confusion and new findings
Source: BMC Public Health. 2024 Jan 15;24:180. doi: 10.1186/s12889-023-17582-8 (PMC10789005; doi:10.1186/s12889-023-17582-8)
Supplement: Supplementary file 6 — Additional file 6. [file 12889_2023_17582_MOESM6_ESM.pdf]

Supplement Figure 6.The association between Na:K ratio and all-cause mortality with T2 Na:K ratio compared to T1 Na:K ratio in each subgroups.

subgroup	HR (95% CI)	P for interaction
Sex		0.78
Male	1.03 (0.79 to 1.34)	
Female	0.93 (0.70 to 1.22)	
Age		0.64
[40,60]	0.93 (0.62 to 1.39)	
[60,80]	0.96 (0.78 to 1.18)	
Race		0.93
Mexican	1.08 (0.73 to 1.61)	
Hispanic	0.77 (0.34 to 1.73)	
White	1.01 (0.81 to 1.27)	
Black	0.71 (0.52 to 0.97)	
Other	1.15 (0.51 to 2.62)	
Education		0.095
below	1.04 (0.81 to 1.32)	
above	0.92 (0.69 to 1.23)	
Diabetes		0.64
No	0.96 (0.76 to 1.20)	
Yes	1.08 (0.80 to 1.46)	
HTN		0.81
No	0.81 (0.58 to 1.13)	
Yes	1.09 (0.88 to 1.36)	
CVD		0.49
No	1.05 (0.83 to 1.33)	
Yes	0.84 (0.63 to 1.10)	
CKD		0.93
No	0.96 (0.78 to 1.18)	
Yes	0.99 (0.69 to 1.42)	
PIR		0.14
Lower	0.94 (0.72 to 1.23)	
Higher	1.00 (0.79 to 1.26)	
BMI		0.72
Normal	1.13 (0.80 to 1.58)	
Overweight	1.00 (0.69 to 1.44)	
Obesity	0.93 (0.73 to 1.20)	
Summary	0.98 (0.81 to 1.20)	

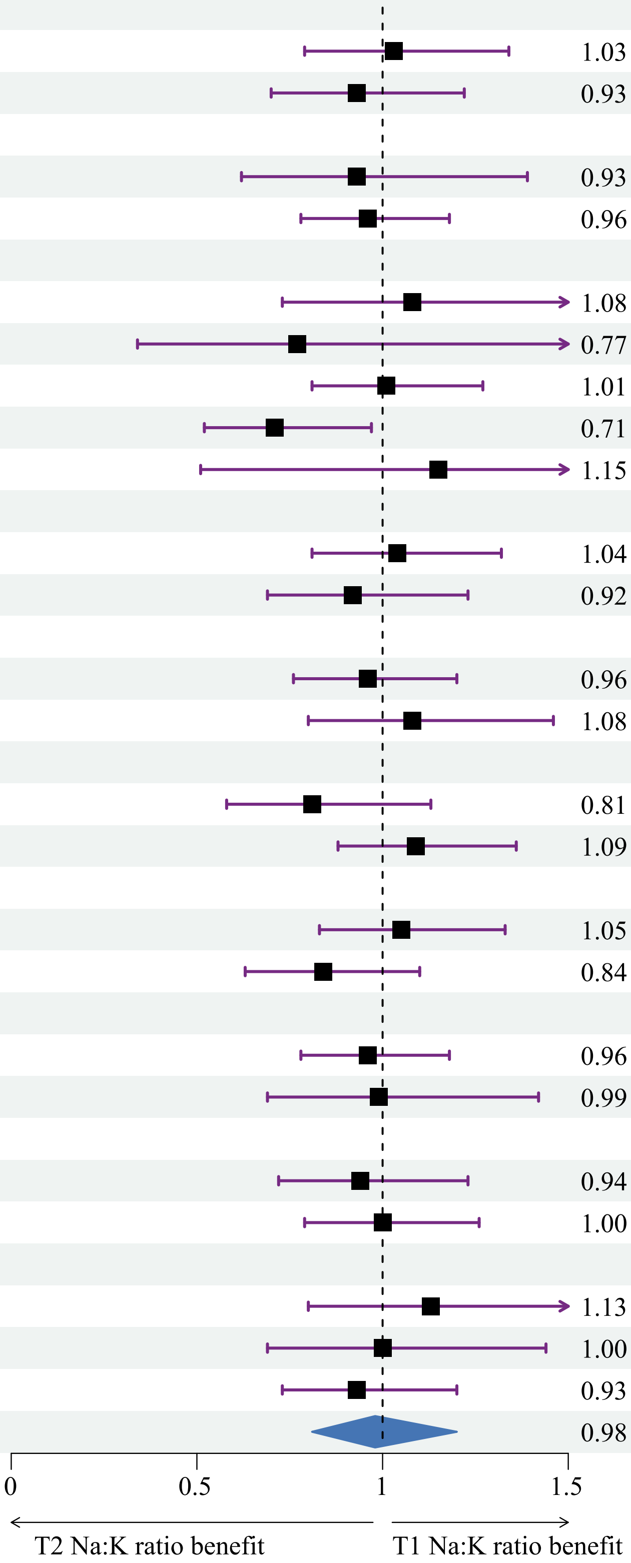

Forest plots of stratified analyses of Na:K ratio and all-cause mortality. all covariates except for the factor defining the subgroup were adjusted . The multicollinearity test was conducted for all variables in the models. There is a certain degree of multicollinearity (VIF>10) present in the populations of Mexico, Spain, and others, which may hinder the extrapolation of this conclusion to these populations.
