# Supplementary material for: Sodium, potassium intake, and all-cause mortality: confusion and new findings
Source: BMC Public Health. 2024 Jan 15;24:180. doi: 10.1186/s12889-023-17582-8 (PMC10789005; doi:10.1186/s12889-023-17582-8)
Supplement: Supplementary file 7 — Additional file 7. [file 12889_2023_17582_MOESM7_ESM.pdf]

Supplement Figure 7.The association between Na:K ratio and all-cause mortality with T3 Na:K ratio compared to T1 Na:K ratio in each subgroups.

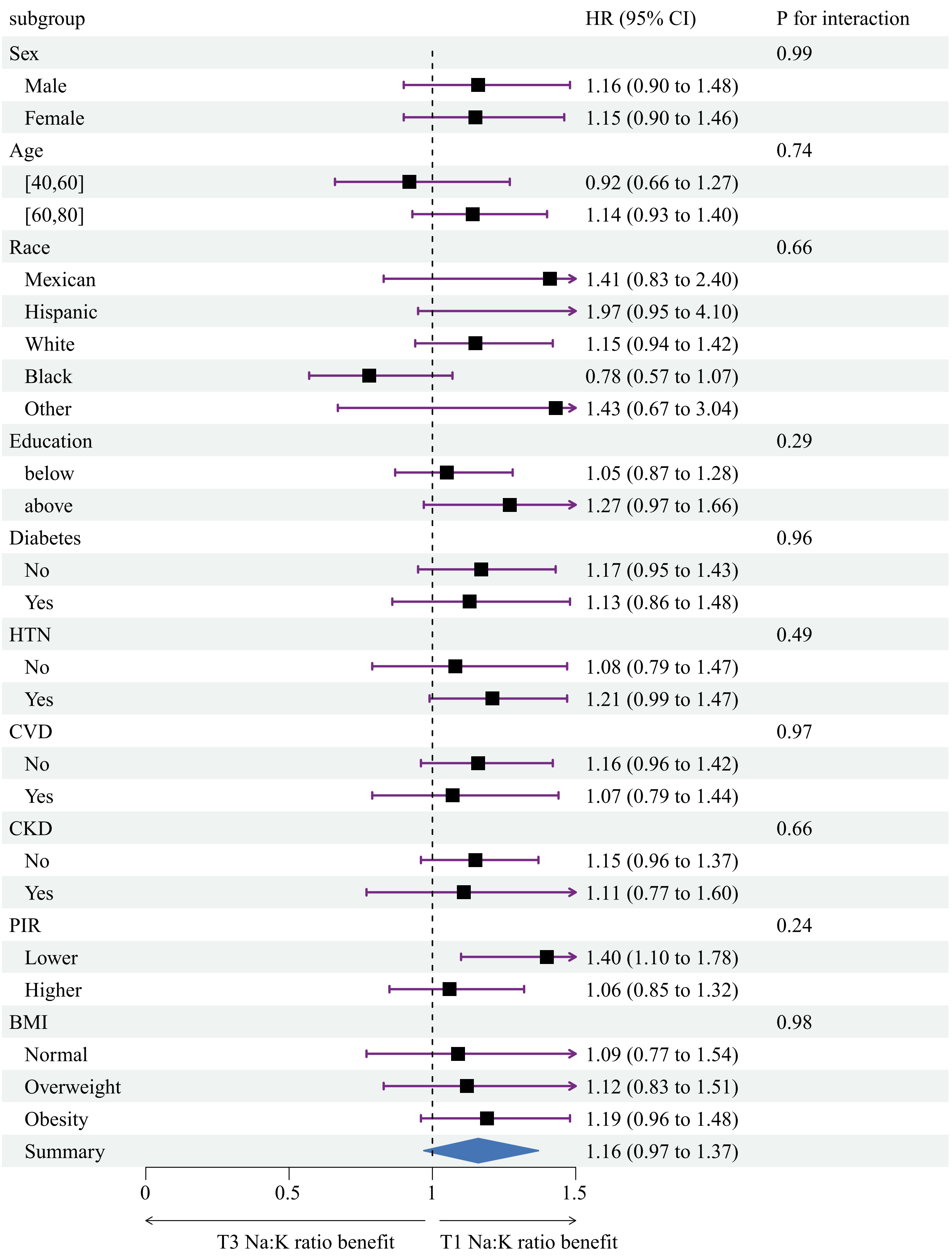

Forest plots of stratified analyses of Na:K ratio and all-cause mortality. all covariates except for the factor defining the subgroup were adjusted . The multicollinearity test was conducted for all variables in the models. There is a certain degree of multicollinearity (VIF>10) present in the populations of Mexico, Spain, and others, which may hinder the extrapolation of this conclusion to these populations.
