# Supplementary material for: Sodium, potassium intake, and all-cause mortality: confusion and new findings
Source: BMC Public Health. 2024 Jan 15;24:180. doi: 10.1186/s12889-023-17582-8 (PMC10789005; doi:10.1186/s12889-023-17582-8)
Supplement: Supplementary file 8 — Additional file 8. [file 12889_2023_17582_MOESM8_ESM.pdf]

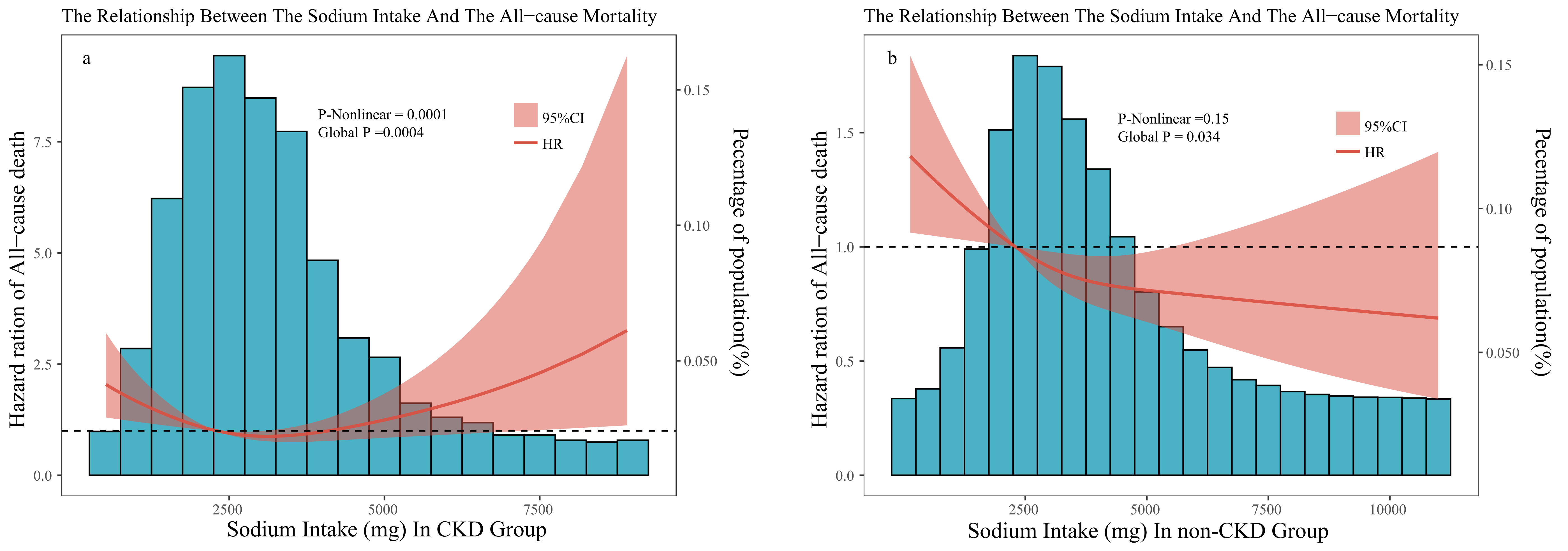

The Relationship Between The Sodium Intake And The All−cause Mortality
a
P-Nonlinear = 0.0001
Global P =0.0004
95%CI
HR
Hazard ration of All−cause death
Pecentage of population(%)
0.0
2.5
5.0
7.5
0.050
0.10
0.15
2500
5000
7500
Sodium Intake (mg) In CKD Group
The Relationship Between The Sodium Intake And The All−cause Mortality
b
P-Nonlinear =0.15
Global P = 0.034
95%CI
HR
Hazard ration of All−cause death
Pecentage of population(%)
0.0
0.5
1.0
1.5
0.050
0.10
0.15
2500
5000
7500
10000
Sodium Intake (mg) In non-CKD Group
