# Supplementary material for: Sodium, potassium intake, and all-cause mortality: confusion and new findings
Source: BMC Public Health. 2024 Jan 15;24:180. doi: 10.1186/s12889-023-17582-8 (PMC10789005; doi:10.1186/s12889-023-17582-8)
Supplement: Supplementary file 9 — Additional file 9. [file 12889_2023_17582_MOESM9_ESM.docx]

Supplement Table 1. Baseline characteristics of participants with complex weighting

| **Characteristic** | **Participants**  , N = 13,499,635^1^ | **Participants**  **with no missing**  , N = 11,348,771^1^ | **p-value**^2^ |
| --- | --- | --- | --- |
| Sex |  |  | <0.001 |
| male | 6,666,435 (49%) | 5,701,668 (50%) |  |
| female | 6,833,200 (51%) | 5,647,103 (50%) |  |
| Age(years) | 56 (11) | 56 (10) | 0.6 |
| Race |  |  | <0.001 |
| Mexican American | 842,107 (6.2%) | 665,390 (5.9%) |  |
| Other Hispanic | 572,470 (4.2%) | 431,714 (3.8%) |  |
| Non-Hispanic White | 9,929,376 (74%) | 8,607,932 (76%) |  |
| Non-Hispanic Black | 1,217,507 (9.0%) | 924,886 (8.1%) |  |
| Other | 938,174 (6.9%) | 718,850 (6.3%) |  |
| Education |  |  | <0.001 |
| No | 5,256,690 (39%) | 4,310,533 (38%) |  |
| Yes | 8,239,484 (61%) | 7,038,238 (62%) |  |
| PIR |  |  | <0.001 |
| Lower | 2,103,223 (17%) | 1,812,801 (16%) |  |
| Higher | 10,498,423 (83%) | 9,535,970 (84%) |  |
| Smoking Now |  |  | 0.007 |
| No | 6,916,992 (51%) | 5,760,664 (51%) |  |
| Yes | 6,535,370 (49%) | 5,588,107 (49%) |  |
| Drink Levels |  |  | <0.001 |
| No-drinker | 3,534,519 (28%) | 3,059,274 (27%) |  |
| 1-10 drinks/month | 6,784,001 (53%) | 6,046,148 (53%) |  |
| 10+ drinks/month | 2,470,752 (19%) | 2,243,349 (20%) |  |
| Hypertension |  |  | 0.12 |
| No | 6,870,268 (52%) | 5,905,237 (52%) |  |
| Yes | 6,436,257 (48%) | 5,443,534 (48%) |  |
| Diabetes |  |  | 0.5 |
| No | 11,057,850 (82%) | 9,309,448 (82%) |  |
| Yes | 2,441,785 (18%) | 2,039,323 (18%) |  |
| CVD |  |  | 0.3 |
| No | 11,965,105 (89%) | 10,075,870 (89%) |  |
| Yes | 1,534,529 (11%) | 1,272,901 (11%) |  |
| Body mass index (kg/m2) | 29 (7) | 30 (7) | 0.056 |
| eGFR(ml/min) | 89 (18) | 89 (18) | 0.10 |
| Physical activity |  |  | <0.001 |
| No | 3,037,832 (23%) | 2,470,112 (22%) |  |
| Yes | 10,461,803 (77%) | 8,878,659 (78%) |  |
| Marriage |  |  | 0.063 |
| single | 4,015,852 (30%) | 3,329,778 (29%) |  |
| couple | 9,477,526 (70%) | 8,018,993 (71%) |  |
| Sodium Intake(mg) | 3,412 (1,429) | 3,448 (1,424) | <0.001 |
| Dietary calories (kcal) | 2,067 (792) | 2,089 (783) | <0.001 |
| Potassium Intake(mg) | 2,778 (1,087) | 2,799 (1,084) | <0.001 |
| ^1^n (%); Mean (SD) | | | |
| ^2^chi-squared test with Rao & Scott's second-order correction; Wilcoxon rank-sum test for complex survey samples | | | |
