# Supplementary material for: Sodium, potassium intake, and all-cause mortality: confusion and new findings
Source: BMC Public Health. 2024 Jan 15;24:180. doi: 10.1186/s12889-023-17582-8 (PMC10789005; doi:10.1186/s12889-023-17582-8)
Supplement: Supplementary file 10 — Additional file 10. [file 12889_2023_17582_MOESM10_ESM.docx]

Supplement Table 2. Baseline characteristics of participants according to daily sodium intake.

| **Sodium Intake** | | | | | |
| --- | --- | --- | --- | --- | --- |
| **Characteristic** | **Overall**, N = 13,855^1^ | **Low Intake**,  N = 3,435^1^ | **Normal Intake**,  N = 8,186^1^ | **High Intake**,  N = 2,234^1^ | **p-value**^2^ |
| Sex |  |  |  |  | <0.001 |
| Female | 6,633 (48%) | 2,248 (65%) | 3,946 (48%) | 439 (20%) |  |
| Male | 7,222 (52%) | 1,187 (35%) | 4,240 (52%) | 1,795 (80%) |  |
| Age(years) | 58 (49, 67) | 61 (51, 69) | 58 (49, 67) | 54 (46, 62) | <0.001 |
| Race |  |  |  |  | <0.001 |
| Mexican American | 2,075 (15%) | 629 (18%) | 1,133 (14%) | 313 (14%) |  |
| Other Hispanic | 1,145 (8.3%) | 363 (11%) | 648 (7.9%) | 134 (6.0%) |  |
| Non-Hispanic White | 6,871 (50%) | 1,444 (42%) | 4,234 (52%) | 1,193 (53%) |  |
| Non-Hispanic Black | 2,603 (19%) | 769 (22%) | 1,485 (18%) | 349 (16%) |  |
| Other | 1,161 (8.4%) | 230 (6.7%) | 686 (8.4%) | 245 (11%) |  |
| Marriage |  |  |  |  | <0.001 |
| Couple | 9,185 (66%) | 2,081 (61%) | 5,519 (67%) | 1,585 (71%) |  |
| Single | 4,670 (34%) | 1,354 (39%) | 2,667 (33%) | 649 (29%) |  |
| PIR |  |  |  |  | <0.001 |
| Lower | 3,725 (27%) | 1,194 (35%) | 2,020 (25%) | 511 (23%) |  |
| Higher | 10,130 (73%) | 2,241 (65%) | 6,166 (75%) | 1,723 (77%) |  |
| Smoking Now |  |  |  |  | <0.001 |
| No | 6,839 (49%) | 1,781 (52%) | 4,033 (49%) | 1,025 (46%) |  |
| Yes | 7,016 (51%) | 1,654 (48%) | 4,153 (51%) | 1,209 (54%) |  |
| Drink |  |  |  |  | <0.001 |
| No-drinker | 4,438 (32%) | 1,441 (42%) | 2,463 (30%) | 534 (24%) |  |
| 1-10 drinks/month | 7,202 (52%) | 1,614 (47%) | 4,316 (53%) | 1,272 (57%) |  |
| 10 drinks/month | 2,215 (16%) | 380 (11%) | 1,407 (17%) | 428 (19%) |  |
| Hypertension |  |  |  |  | <0.001 |
| No | 6,436 (46%) | 1,462 (43%) | 3,843 (47%) | 1,131 (51%) |  |
| Yes | 7,419 (54%) | 1,973 (57%) | 4,343 (53%) | 1,103 (49%) |  |
| Diabetes |  |  |  |  | <0.001 |
| No | 10,569 (76%) | 2,538 (74%) | 6,287 (77%) | 1,744 (78%) |  |
| Yes | 3,286 (24%) | 897 (26%) | 1,899 (23%) | 490 (22%) |  |
| CVD |  |  |  |  | <0.001 |
| No | 11,963 (86%) | 2,901 (84%) | 7,064 (86%) | 1,998 (89%) |  |
| Yes | 1,892 (14%) | 534 (16%) | 1,122 (14%) | 236 (11%) |  |
| Body mass index (kg/m2) | 30 (7) | 29 (7) | 30 (7) | 30 (7) | 0.004 |
| eGFR(ml/min) | 87 (19) | 84 (21) | 87 (19) | 91 (17) | <0.001 |
| Physical activity |  |  |  |  | <0.001 |
| No | 3,754 (27%) | 1,112 (32%) | 2,121 (26%) | 521 (23%) |  |
| Yes | 10,101 (73%) | 2,323 (68%) | 6,065 (74%) | 1,713 (77%) |  |
| Education |  |  |  |  | <0.001 |
| College or AA degree below | 6,607 (48%) | 1,973 (57%) | 3,680 (45%) | 954 (43%) |  |
| College or AA degree above | 7,248 (52%) | 1,462 (43%) | 4,506 (55%) | 1,280 (57%) |  |
| Sodium Intake(mg) | 3306 (1414) | 1758 (407) | 3,294 (628) | 5728 (1070) | <0.001 |
| Dietary calories (kcal) | 2008 (777) | 1,310 (414) | 2020 (528) | 3033 (809) | <0.001 |
| Potassium Intake(mg) | 2683 (1062) | 1926 (775) | 2701 (850) | 3033 (809) | <0.001 |
| ^1^n (%); Mean (SD) | | | | | |
| ^2^Pearson's Chi-squared test; Kruskal-Wallis rank sum test | | | | | |
