# Supplementary material for: Sodium, potassium intake, and all-cause mortality: confusion and new findings
Source: BMC Public Health. 2024 Jan 15;24:180. doi: 10.1186/s12889-023-17582-8 (PMC10789005; doi:10.1186/s12889-023-17582-8)
Supplement: Supplementary file 11 — Additional file 11. [file 12889_2023_17582_MOESM11_ESM.docx]

Supplement Table 3. Baseline characteristics of participants according to daily Potassium Intake

| **Potassium Intake** | | | | | |
| --- | --- | --- | --- | --- | --- |
| **Characteristic** | **Overall**, N = 13,855^1^ | **T1**,  N = 4,622^1^ | **T2**,  N = 4,625^1^ | **T3**,  N = 4,608^1^ | **p-value**^2^ |
| Sex |  |  |  |  | <0.001 |
| Female | 6,633 (48%) | 2,872 (62%) | 2,344 (51%) | 1,417 (31%) |  |
| Male | 7,222 (52%) | 1,750 (38%) | 2,281 (49%) | 3,191 (69%) |  |
| Age(years) | 58 (49, 67) | 60 (49, 68) | 59 (49, 67) | 57 (48, 66) | <0.001 |
| Race |  |  |  |  | <0.001 |
| Mexican American | 2,075 (15%) | 690 (15%) | 718 (16%) | 667 (14%) |  |
| Other Hispanic | 1,145 (8.3%) | 418 (9.0%) | 397 (8.6%) | 330 (7.2%) |  |
| Non-Hispanic White | 6,871 (50%) | 1,909 (41%) | 2,337 (51%) | 2,625 (57%) |  |
| Non-Hispanic Black | 2,603 (19%) | 1,245 (27%) | 786 (17%) | 572 (12%) |  |
| Other | 1,161 (8.4%) | 360 (7.8%) | 387 (8.4%) | 414 (9.0%) |  |
| Marriage |  |  |  |  | <0.001 |
| Couple | 9,185 (66%) | 2,777 (60%) | 3,091 (67%) | 3,317 (72%) |  |
| Single | 4,670 (34%) | 1,845 (40%) | 1,534 (33%) | 1,291 (28%) |  |
| PIR |  |  |  |  | <0.001 |
| Lower | 3,725 (27%) | 1,580 (34%) | 1,147 (25%) | 998 (22%) |  |
| Higher | 10,130 (73%) | 3,042 (66%) | 3,478 (75%) | 3,610 (78%) |  |
| Smoking Now |  |  |  |  | 0.003 |
| No | 6,839 (49%) | 2,337 (51%) | 2,322 (50%) | 2,180 (47%) |  |
| Yes | 7,016 (51%) | 2,285 (49%) | 2,303 (50%) | 2,428 (53%) |  |
| Drink |  |  |  |  | <0.001 |
| No-drinker | 4,438 (32%) | 1,862 (40%) | 1,465 (32%) | 1,111 (24%) |  |
| 1-10 drinks/month | 7,202 (52%) | 2,262 (49%) | 2,433 (53%) | 2,507 (54%) |  |
| 10 drinks/month | 2,215 (16%) | 498 (11%) | 727 (16%) | 990 (21%) |  |
| Hypertension |  |  |  |  | <0.001 |
| No | 6,436 (46%) | 1,902 (41%) | 2,183 (47%) | 2,351 (51%) |  |
| Yes | 7,419 (54%) | 2,720 (59%) | 2,442 (53%) | 2,257 (49%) |  |
| Diabetes |  |  |  |  | <0.001 |
| No | 10,569 (76%) | 3,330 (72%) | 3,522 (76%) | 3,717 (81%) |  |
| Yes | 3,286 (24%) | 1,292 (28%) | 1,103 (24%) | 891 (19%) |  |
| CVD |  |  |  |  | <0.001 |
| No | 11,963 (86%) | 3,873 (84%) | 4,014 (87%) | 4,076 (88%) |  |
| Yes | 1,892 (14%) | 749 (16%) | 611 (13%) | 532 (12%) |  |
| Body mass index (kg/m2) | 30 (7) | 30 (7) | 30 (7) | 29 (6) | <0.001 |
| eGFR(ml/min) | 87 (19) | 85 (21) | 87 (19) | 89 (17) | <0.001 |
| Physical activity |  |  |  |  | <0.001 |
| No | 3,754 (27%) | 1,578 (34%) | 1,233 (27%) | 943 (20%) |  |
| Yes | 10,101 (73%) | 3,044 (66%) | 3,392 (73%) | 3,665 (80%) |  |
| Education |  |  |  |  | <0.001 |
| College or AA degree below | 6,607 (48%) | 2,652 (57%) | 2,131 (46%) | 1,824 (40%) |  |
| College or AA degree above | 7,248 (52%) | 1,970 (43%) | 2,494 (54%) | 2,784 (60%) |  |
| ^1^n (%); Mean (SD) | | | | | |
| ^2^Pearson's Chi-squared test; Kruskal-Wallis rank sum test | | | | | |
